# Stool and Plasma Metabolites and APOE status in the Boston Puerto Rican Health Study

**DOI:** 10.1002/alz70856_105393

**Published:** 2026-01-08

**Authors:** Natalia Palacios

**Affiliations:** ^1^ Department of Public Health, University of Massachusetts Lowell, Lowell, MA, USA

## Abstract

**Background:**

The Apolipoprotein E (APOE) *e4* variant is the strongest known genetic risk factor for dementia and Alzheimer's disease. It has been associated with alterations in glucose and lipid metabolism, increased risk of diabetes, metabolic syndrome, and dysbiosis of the gut microbiota. To our knowledge, no studies have concurrently examined untargeted metabolites in both stool and plasma with APOE‐e4 genotype. Evidence is especially lacking in Latinos/Hispanics who have unique metabolic and microbiome profiles and are at an increased risk of dementia.

**Methods:**

Our analysis focused on 209 Boston Puerto Rican Health Study participants (median age 68 y) who provided concurrent stool and blood samples for untargeted metabolomic profiling (Metabolon, Inc) and had APOE *e4* genotyping. Multivariate analyses in MaAsLin2 were used to identify stool and plasma metabolites associated with APOE status (carrier of at least one APOE *e4* allele, vs non‐carrier). Overrepresentation analysis (ORA) was used to identify metabolite sets enriched in those with APOE *e4* genotype.

**Results:**

In plasma, ORA identified overrepresentation of Primary Bile Acid Metabolism metabolites among associations with APOE *e4* (pFDR = 0.0205). Consistently, MaAsLi2 identified several bile acid plasma metabolites associated with APOE e4, such as glycocholate (pFDR = 0.047), glycochenodeoxycholate‐3‐sulfate (pFDR = 0.11), glycohyocholate (pFDR = 0.18), and glycochenodeoxyhcholate (FDR q = 0.21). In stool, ORA identified dipeptide (pFDR = 0.0067) as overrepresented among those with APOE *e4*; no metabolites met the pFDR < 0.25 threshold in MaAsLin2 analyses.

**Conclusion:**

Our findings are consistent with prior reports highlighted dysregulation in bile acid metabolism in APOE *e4*carriers. More work is needed to understand the role of APOEe4, stool and serum metabolome, bile acid metabolism in cognitive function and and dementia risk.

